# Effectiveness of Different Soft Acaricides against Honey Bee Ectoparasitic Mite *Varroa destructor* (Acari: Varroidae)

**DOI:** 10.3390/insects12111032

**Published:** 2021-11-17

**Authors:** Ziyad Abdul Qadir, Atif Idrees, Rashid Mahmood, Ghulam Sarwar, Muhammad Abu Bakar, Saboor Ahmad, Muhammad Mohsin Raza, Jun Li

**Affiliations:** 1 Honeybee Research Institute, National Agricultural Research Centre, Park Road, Islamabad 45500, Pakistan; zaqadir@parc.gov.pk (Z.A.Q.); directorhbri@parc.gov.pk (R.M.); sarwar1970@parc.gov.pk (G.S.); 2 Department of Entomology and Wildlife Ecology, University of Delaware, Newark, DE 19716, USA; 3 Guangdong Key Laboratory of Animal Conservation and Resource Utilization, Guangdong Public Laboratory of Wild Animal Conservation and Utilization, Institute of Zoology, Guangdong Academy of Sciences, Guangzhou 510260, China; atif_entomologist@yahoo.com; 4 Department of Entomology, University College of Agriculture, University of Sargodha, Sargodha 40100, Pakistan; mabubakar_26@yahoo.com; 5 Institute of Apicultural Research/Key Laboratory of Pollinating Insect Biology, Ministry of Agriculture, Chinese Academy of Agricultural Sciences, Beijing 100081, China; 2018y90100172@caas.cn; 6 The Joint Centre for Excellence in Environmental Intelligence, University of Exeter, Exeter EX4 4QF, UK; m.r.raza@exeter.ac.uk

**Keywords:** honey bee, formic acid, oxalic acid, thymol, *Varroa destructor*

## Abstract

**Simple Summary:**

Over the past few decades, the ectoparasitic mite *Varroa destructor* has been a significant threat to managed honey bee (*Apis mellifera*) colonies worldwide. Many control methods, including application of synthetic acaricides, have been adopted to control the infestation of varroa mites in honey bee colonies. Synthetic acaricides such as coumaphos and fluvalinate are only effective in reducing susceptible mites. Besides, synthetic acaricides pose multiple threats to honey bee colonies and the environment, necessitating their alteration with non-synthetic options. Naturally occurring compounds are considered an essential alternative control measure for varroa mites. Natural acaricides are derived from plants that contain essential oils or organic acids. The current study investigated the efficacy of formic acid, oxalic acid, and thymol in the control of Varroa mites. These soft acaricides were applied at various concentrations/quantities. Formic acid, oxalic acid, and thymol were all effective at lowering mite population levels. Formic acid, oxalic acid, and thymol can be used in an integrated management plan to control varroa mite populations. This scientific-based information can be shared with the beekeeping community of Pakistan and elsewhere, which will be helpful in managing this parasite that often affects honey bee productivity.

**Abstract:**

Honey bees (*Apis mellifera*) are essential for their products—honey, royal jelly, pollen, propolis and beeswax. They are also indispensable because they support ecosystems with their pollination services. However, the production and functions of honey bees are hindered by the arthropod pest *Varroa destructor*, which attacks bees through its feeding activities. Efforts to control varroa mites have been made through the development of various synthetic pesticide groups, but have had limited success because the mites developed resistance and some of these pesticides are harmful to bees. Branded pesticides are rarely used in Pakistan, as beekeepers utilize acaricides from unknown sources. There is a need to create awareness of available naturally occurring acaricides that may serve as an alternative to synthetic acaricides. Although some naturally occurring compounds are considered toxic to the environment, the soft acaricides oxalic acid, thymol, and formic acid 65% are usually safe for honey bee colonies and beekeepers, when handled appropriately. The current study investigated the effectiveness of formic acid (10, 15, and 20 mL/hive), oxalic acid (4.2, 3.2, and 2.1%/hive), and thymol (6, 4, and 2 g/hive) in controlling mite infestation. The results indicated that all treatments significantly reduced the mite population (*p* < 0.05). The average efficacies of oxalic acid at 3.2% (94.84% ± 0.34) and 4.2% (92.68% ± 0.37) were significantly higher than those of the other treatments. The lowest efficacy was recorded in formic acid 65% at 10 mL (54.13%). Overall, the results indicated that soft acaricides—such as oxalic acid at 3.2% and 4.2% concentrations—are very effective at controlling varroa mites and can be used in broodless conditions without side effects.

## 1. Introduction

Honey bees, *Apis mellifera* (L.) (Hymenoptera: Apidae), play an essential role in agriculture by producing commodities, such as honey, propolis, royal jelly, bee pollen, bee venom, and beeswax [1]. Additionally, it is the most crucial eusocial insect, having benefited humankind for medicinal and nutritional purposes for thousands of years [2]. In 1977, grand-scale beekeeping experiments were initiated at the Honey bee Research Institute, under the National Agricultural Research Centre, Islamabad, Pakistan [3]. *Apis mellifera* was introduced in Pakistan to build an industry capable of producing substantial surplus honey for export as a cash crop [4]. In 2010, Pakistani beekeeping was being practiced by 27,000 families that were rearing ~400,000 beehives of *A. mellifera* and were financially benefitting from this profession [4,5]. Pakistan has also exported 4179.953 metric tons of honey valued at U.S. $9.8 million in 2020 [6].

Different insect pests and microorganisms attack honey bees around the world, including in Pakistan [7]. The acarine mite *Varroa destructor* is one of the most serious problems for *A. mellifera* [8]. It has affected both the honey bee industry and honey bee research activities since the dawn of apiculture [9]. *Varroa destructor* is considered the primary cause of the global decline in honey bees, resulting in huge colony losses [10,11].

The mite attacks the occidental bees in Pakistan [3]. After the introduction and successful domestication of the occidental bee, the mite became a serious pest of the bee and destroyed a considerable number of colonies within a few years [12]. In Pakistan, in 2002, two ectoparasitic mites—*V. destructor* and *Tropilaelaps clareae*—reduced honey production by up to 50% [13]. Previously, it was thought that varroa mites fed on the hemolymph of adult honey bees, sealed brood, and larvae, but recently, researchers have found that mites directly feed on body fat tissues and cellular components of immature and mature bees [14].

It is certain that, if appropriate control measures are not practiced to control mites on *A. mellifera* colonies, more damage (such as complete colony loss) can be anticipated [15,16]. In Pakistan, there exist neither honey bee regulatory bodies, nor well-regulated pesticides [17]. Thus, no checks and balances exist on the usage of pesticides, as some beekeepers often use acaricides such as formic acid from unknown sources while others rarely use branded soft acaricides from scientific stores. Environmentally safe options are needed to control various insect pests [18,19,20,21,22]. Different hard acaricides have been used to control this mite but have resulted in increasing mite resistance against these hard acaricides, and ultimately a reduction in their effectiveness [23]. For example, 24 years ago, *V. destructor* developed resistance to fluvalinate [24]. In addition, certain mite populations have established cross-resistance to different pesticide groups, i.e., formamidine (amitraz), organophosphates (coumaphos), and pyrethroids (acrinathin and flumethrin) [25,26,27,28,29]. These pesticides also pose a threat to bees by harming them when the bees are exposed to multiple compounds stored in wax. Accumulation of these hard acaricides in wax can create resistance in mites that are present in sealed cells, making the mites challenging to control [9]. Thus, these acaricides pose a threat to the beekeeping industry and pollute honey and other honey bee products [9]. Multiple soft acaricides are thought to play a critical role in the management of varroa mites. Soft acaricides are natural compounds of plant origin that contain essential oils or organic acids with pesticidal properties [30,31]. They are low-environmental-impact acaricides believed to be harmless to human health when handled appropriately [32]. Hard acaricides on the other hand usually contain synthetic and high-environmental-impact constituents proven to be effective against mites, but can on the other hand affect honey bees and other hive products [9].

Formic acid, lactic acid, oxalic acid, and thymol are organic acids that are used to control varroa and characterize the basis of natural compounds [9]. These natural compounds have various effects against mites. Formic acid interferes with the respiratory system of both adult mites and sealed brood cells [33]. In addition, most of these soft acaricides are water soluble and volatile, have a low risk of accumulation in bee products, and have lower residue levels [9]. Furthermore, they are natural constituents of honey [9]. Thus, they are less likely to contaminate and affect both bees and honey bee products [34,35,36,37]. Repeated treatments have a low probability of developing resistance [9]. These soft acaricides are season dependent; colony condition (brood or broodless) is also taken into account before applying these soft acaricides [9]. Similar to thymol, it was reported by Baggio et al. [38] to not use powdered thymol on weak colonies at high temperatures (>27–30 °C). Oxalic acid (OA) is highly effective in treating colonies without brood [39,40].

Varroa mites are becoming a serious threat to the beekeeping industry worldwide. This study aimed to manage varroa mites by using different soft acaricides (formic acid, oxalic acid, and thymol) in respective colonies; changes in honey production were also measured. This study also evaluated whether these chemicals are environmentally friendly, nonresistant, safe for human health, and safe for bee health. The objective of the study was to determine the effectiveness of soft acaricides both at the group level and among different concentration/quantity levels. Similarly, we compared the honey yields of all treatment groups at both the group and different concentration/quantity levels.

## 2. Materials and Methods

### 2.1. Study Site

The present study was carried out at the apiary of the Honeybee Research Institute, National Agricultural Research Centre, Islamabad, Pakistan, during the winter season (December to January). In December, the maximum and minimum temperature was 19.3, and 4.13 °C, respectively; the wind speed was 10.56 Km/day; 18.19 mm rainfall, and the average relative humidity was 76%. In January, the maximum and minimum temperature was 17.2 and 3.85 °C; the wind speed was 15.88 Km/day; rainfall was 41.64 mm, and the average relative humidity was 82%. This study was conducted on honey bee *A. mellifera* colonies that were naturally infested with varroa mites.

### 2.2. Honey Bee Colonies

Before initializing the experiments, 90 honey bee colonies of *A. mellifera* were selected to record the natural varroa mite fall. All of the colonies were housed in standard Langstroth boxes. A total of 45 colonies were screened out of the experiment to have colonies with the following characteristics: a honey bee population roughly equal to that of the other colonies, queen right and possess a mite infestation with an economic threshold level of >10 adult mites [8]. Furthermore, all experimental colonies were fully developed and productive. These were monitored prior to experimentation and had an average of 8 ± 2 brood combs in their brood chambers [41]. Five colonies were in each treatment group.

### 2.3. Soft Acaricide Treatments

Three soft acaricides, each at three concentration levels or quantities, were used in the study. The acaricides were produced by BDH laboratory supplies, England. Both inter- and intra-comparisons were conducted among the nine treatment groups. Soft acaricides used in the experiments were as follow:

#### 2.3.1. Formic Acid (AnalaR 98/100% ‘Safe-Break’)

Formic acid (65%) at different quantities (10, 15, and 20 mL/hive) was used in this experimental study. Formic acid treatments were applied by pouring 10, 15, and 20 m of 65% formic acid on a piece of cardboard (7.5″ × 5.5″) placed inside the wire meshed tray inserted above the bottom board from the backside of the hive [42]. Formic acid solution was applied weekly to all colonies over the course of one month for four treatments.

#### 2.3.2. Oxalic Acid (AnalaR)

Three different oxalic acid concentrations (4.2%, 3.2%, and 2.1%) mixed with sugar syrup were applied as treatments. To attain 4.2%, 3.2%, and 2.1% oxalic acid solutions, 100, 75, and 50 g of oxalic acid dehydrate was mixed with 1 L of sugar water (1:1) [43]. Treatments were applied only to frame spaces that contained bees. The 5-mL mixture was trickled directly on the adult bees in between two frames using a syringe as recommended (i.e., ~50 mL/colony) [39,40]. All groups received four doses of oxalic acid solution after a one-week interval.

#### 2.3.3. Thymol (GPR)

Three different thymol concentrations (6, 4, and 2 g) were applied as treatments. Finely ground thymol was placed in petri dishes (80 mm) on top of the brood frame chambers under the top covers of the honey bee colonies [44]. Each treatment was applied after a one-week interval.

### 2.4. Mite Collection

To assess the adult mite population, mite collection trays were sandwiched between bottom boards from the backside of the beehive with wire screens installed above them to prevent the bees from removing the dead (or live) mites along with the debris [42,44]. Apart from checking for mite infestation levels, mite collection trays also improve treatment effectiveness. Dead fallen mites were examined after 7, 14, 21, and 28 days by counting dead fallen mites on the mite collection trays. To calculate the effectiveness of the applied treatments, Manhao^TM^, Sichuan Pengshan Wangshi Animal Health Co., Ltd, (Chengdu, China) (fluvalinate) strips were applied to all of the treated colonies to knock down the remaining mites and evaluate the total mite population. Manhao^TM^ (fluvalinate) strips were inserted into each hive immediately following the fourth week of each soft acaricide treatment. Manhao^TM^ (fluvalinate) strips were removed after 28 days, and all the dropped mites were counted on the mite collection trays [45]. Effectiveness of each soft acaricides treatment was calculated separately by using the following efficacy formula [46,47].

$$Efficacy\;\left(\%\right)=\frac{Number\;of\;mites\;fallen\;during\;treatment\;with\;each\;soft\;chemical\;}{Total\;number\;of\;fallen\;mites\;during\;soft\;chemical\;and\;Apistan®\;treatment}\;\times \;100$$

### 2.5. Honey Yield

Honey was harvested after the experiment with the help of a manually operated honey harvester, and the honey yield of treated colonies was recorded. Honey production was measured by taking the weight of each hive body used for honey collection before and after the honey extraction process. The weight difference was considered as the amount of harvestable honey [48].

### 2.6. Statistical Analysis

We used linear mixed effect models to evaluate the impact of each treatment (3 groups) and their concentrations/quantities (9 treatments) on mites in R software version 4.0.2 (R CoreTeam 2019, Vienna, Austria). Each response variable was separately analyzed. R packages including “*lme4*” [49] and “*lmerTest*” [50] were used for fitting the mixed models. In the case of efficacy and honey yield data, treatments were treated as fixed effects, while replications were treated as random effects. Model quality was evaluated based on scaled residuals simulated from the fitted model provided by the *SimulateResiduals* function of the DHARMa R package [51].

Mite data were not normally distributed; thus, we took the natural log to fulfill the normality and homoscedasticity assumptions. Means of significant interactions were compared using the Tukey HSD test at the 5% level of significance using the emmeans package [52]. For unique lettering in the means comparison, we used the Scott Knott clustering method provided in the ScottKnott package of R [53]. Finally, bar charts of significant means were plotted using the ggplot2 package [54] in R.

## 3. Results

### 3.1. Treatment Effectiveness among Major Groups (Formic Acid 65%, Oxalic Acid, and Thymol)

**Figure 1:**
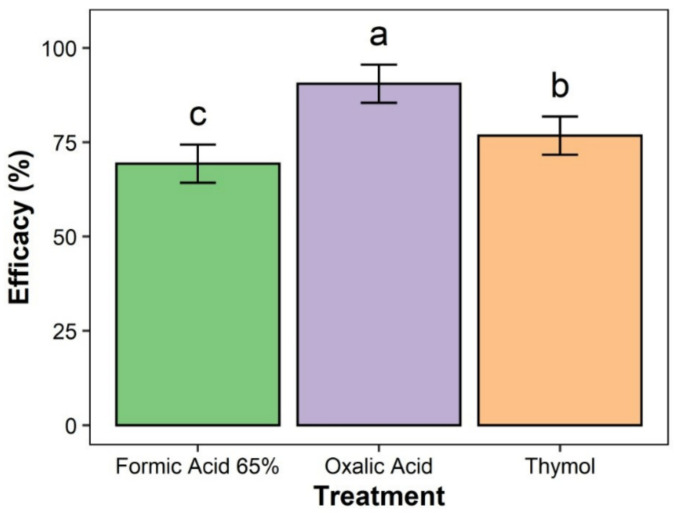
Efficacy of different treatment groups. Bars with the same letters are not significantly different (*p* > 0.05); the error bars represent the confidence interval (95%).

The results revealed that the three major treatments, formic acid 65%, oxalic acid, and thymol, were significantly different in efficacy (%) (*p* < 0.05) (Figure 1). Colonies treated with oxalic acid had the highest efficiency (90.48%), followed by thymol and formic acid 65% (76.74%) (Figure 1).

### 3.2. Treatment Effectiveness within All Concentrations/Quantities

**Figure 2:**
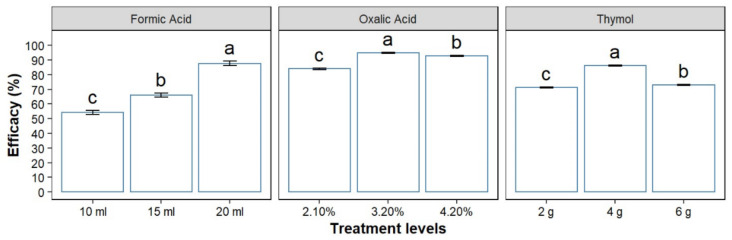
Efficacy of treatments against *Varroa destructor*. Treatments with the same letters are not significantly different; error bars signify confidence intervals (95%).

In terms of concentrations/quantities, honey bee colonies treated with thymol at 4 g, oxalic acid 3.2%, and formic acid 20 mL exhibited the highest efficacy within their respective groups. (Figure 2). While the lowest efficacy was recorded against 2 g of thymol, oxalic acid at 2.10%, and 10-mL formic acid (Figure 2).

All concentrations/quantities within the groups were significantly different in terms of efficacy (Figure 2). Maximum effectiveness was observed in colonies treated with oxalic acid at 3.2% (94.84%), and the lowest efficacy was observed in honey bee colonies treated with formic acid at 65% of 10 mL (54.13%) (Figure 2).

### 3.3. Honey Yield

#### All Concentrations/Quantities

**Figure 3:**
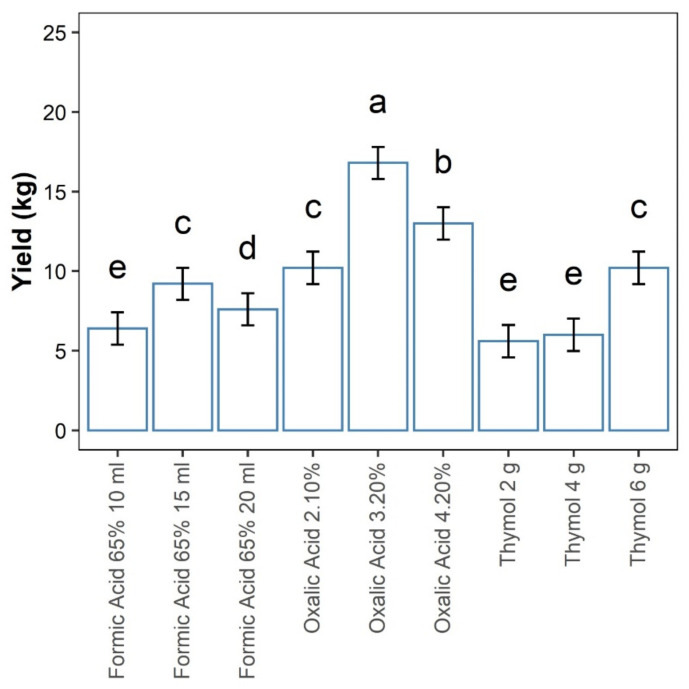
Effect of soft acaricides concentration/quantity on honey yield. Treatments with the same letters are not significantly different; error bars signify confidence intervals (95%).

The highest honey yield was recorded in honey bee colonies treated with oxalic acid (3.20% and 4.20%) at 16.8 kg and 13 kg, respectively (Figure 3). The honey yield was lowest in honey bee colonies treated with 4 g of thymol and 2 g of thymol at 6 kg and 5 kg, respectively (Figure 3).

## 4. Discussion

Mites are economically important pests in honey bee colonies. Soft acaricide treatments are thought to be effective against the ectoparasite mite *V. destructor*. The current study investigated the effectiveness of soft acaricides against *V. destructor.* Our results revealed that oxalic acid is effective against *V. destructor* followed by thymol and formic acid 65% during winter conditions. Acaricides have different effects against *V. destructor* depending on the mode of application, nature of acaricide formulation and environmental conditions [55,56]. Oxalic acid is a winter treatment, thus it is most efficacious at lower temperatures, especially when the brood is not present [57]. Meanwhile, formic acid and thymol are also dependent of different climatic and beekeeping conditions, which cause temperature-dependent effects [9]. Comparison of these soft acaricides needs to be evaluated in various environmental conditions for a more balanced analysis of their efficiency.

In terms of concentration, 3.2% oxalic acid was most effective in reducing *V. destructor* populations, indicating that 3.2% oxalic acid is the optimum concentration for controlling mites. Our results are consistent with Mahmood et al. [44] and Papežíková et al. [55], who reported that 3.2% oxalic acid is a reliable soft acaricide for controlling honey bee mites. Doses higher than 3.2% oxalic acid have also been found to be effective against mites [55]. Generally, the oxalic concentration registered for varroa control in European countries varies between 3.5% and 4.2%, with 4.2% widely used [55]. Our study could not establish why higher doses of oxalic acid (4.2%) did not transform into higher mite mortalities. This was likely because of varying temperatures, which caused a different treatment effect. No bee mortality, queen mortality, or superseding behavior was observed after completing the treatments, which matches the findings of other authors [44].

Our findings revealed that using 4 g of thymol to control *V. destructor* was more effective than 6 or 2 g of thymol. Conversely, it is expected that higher concentrations (in this case, 6 g) would be more effective in controlling varroa mites; this was not the case in the current study. Thymol is most effective during between 20 and 30 °C with effectiveness lost below 15 °C [58]. This may also partly explain the unclear results from the use of thymol as our study, was conducted during winter season. Thymol is a significant component of different commercially available products and is effective in managing ectoparasitic honey bee mites in *A. mellifera* colonies [59,60,61]. Thymol is a potential agent that has shown promising results in controlling ectoparasitic honey bee mites and has no negative impact on bee health [37,62].

It is expected that effectiveness is directly proportional to the dose. However, in the current study the expected dose-response relationship was not observed. Our results could not clearly substantiate the explanations for such observations. The need to explore the reasons for the lack of a dose-response relationship in future studies is critical. Understanding the cause for the lack of a dose-response relationship will not only further our scientific knowledge but it will help provide more useful information to beekeepers.

Our results are in accordance with Rashid et al. [63], revealing that formic acid is least effective in controlling *V. destructor* when tested against different groups. The reduced efficacy of formic acid may be due to the distance between formic acid volatilization and the honey bee-containing combs as the acaricide was only applied on cardboards inserted in the hive. In our study, the cardboard with formic acid application was placed inside the wire meshed tray inserted above the bottom board; this may also affect its efficacy [64,65]. The time of the year and temperature may also have affected its efficacy [65,66,67,68]. Our results contrast those of Mahmood et al. [42], who reported that a formic acid 20-mL concentration is very effective at controlling varroa mites in the winter season after Sider (*Ziziphus mauritiana*) honey harvest. Furthermore, Giusti et al. [69] reported that formic acid had no side effects on larvae, adult bees, and queens and showed an average efficacy greater than 95%. Our findings revealed that 20 mL of 65% formic acid was effective at controlling the honey bee ectoparasitic mite *V. destructor*.

The differences in soft acaricide efficacy in our study could be due to differences in the original number of mites infesting the bee colonies. Our study did not estimate the starting number of mites per colony; thus, it was difficult to conclusively state that the reduction in mite numbers was attributed to soft acaricide treatment. Additionally, studies conducted previously did not provide an estimate of the initial mite population [44,46,70]. Besides, organic acaricides such as those used in the current study may have certain advantages after repeated usage although their efficacy may be inconsistent when compared to synthetic forms [71].

The harvested honey results are in line with Mahmood et al. [47], who reported that honey yield from different treatment groups was significantly different, with the highest honey yield, obtained from oxalic acid, being 3.2%. However, comparison of honey production results across treatments needs to be accompanied by analysis of the colony strength to obtain a more reliable estimate of honey yield. Additionally, evaluating queen bee performance, climate and pasture conditions would further support the honey yield results [55,56]. Although our results indicate differences in the honey yield across the treatments, the contribution of soft acaricides to honey yield production cannot be conclusive as our experimental methods did not take in to account the honey bee populations in the hive after completion of the treatment.

The findings of the present study showed that the soft acaricides—formic acid, oxalic acid, and thymol—are effective natural products against varroa mite populations. The fact that no effects were observed on the honey bee colonies is an indication that the products are safe for the environment. However, further studies are needed to investigate the effectiveness and action mechanisms of soft acaricides against *V. destructor* and their impact on honey bee colony health (queen longevity, effect on worker, and adult brood longevity). Field experiments need to be conducted in varying environmental conditions as the effects of soft acaricides and mite infestation are environmental dependent [58,72]. Controlled experiments involving a known mite population against various concentrations need to be conducted to estimate the lethal concentration (LD_50_). Residual analysis studies to assess residual presence in honey bees, bee wax, and other honey bee products will further confirm the safety of the soft acaricides.

## 5. Conclusions

The soft acaricides (oxalic acid and thymol) used in this experiment at different concentrations were very effective at reducing the damage from the ectoparasitic mite *V. destructor* and controlling its populations in *A. mellifera* colonies without showing any harm to the bees. While different quantities of formic acid 65% showed promising results, these can be included in an integrated mite control program. Beekeepers should use the recommended dosage and registered soft acaricides available in the market while following proper application methods to prevent resistance development in mites. Product use in accordance with the manufacturers directions is essential, as improper use may cause risk to persons or property.

## Data Availability

Not applicable.
